# Complete Genome Sequence of a Human Enterovirus C99 Strain Isolated from a Healthy Child in Yunnan, China, in 2013

**DOI:** 10.1128/MRA.01489-18

**Published:** 2019-04-18

**Authors:** Jie Zhang, Hongbo Liu, Xiaoqin Huang, Hao Sun, Zhaoqing Yang, Shaohui Ma

**Affiliations:** a Institute of Medical Biology, Chinese Academy of Medical Sciences, and Peking Union Medical College, Kunming, People’s Republic of China; b Yunnan Key Laboratory of Vaccine Research Development on Severe Infectious Disease, Kunming, People’s Republic of China; Broad Institute of MIT and Harvard University

## Abstract

The complete genome sequence of a human enterovirus C99 strain isolated from a healthy child in Yunnan, China, in 2013 was determined. The isolate belonged to genotype C, according to phylogenetic and homogeneity analyses.

## ANNOUNCEMENT

Human enterovirus C99 (EV-C99) belongs to Human enterovirus C. EV-C99 is a positive-sense, single-stranded RNA virus. The genome is approximately 7.4 kb, including a 5ʹ untranslated region (UTR), structural polypeptide P1, nonstructural polypeptides P2 and P3, and a 3ʹ UTR. All EV-C99 strains can be classified into three genotypes, A, B, and C (1). EV-C99 strains have been isolated from acute flaccid paralysis (AFP) patients and from oral polio-vaccinated and healthy individuals (1–4). At present, there are very few EV-C99 sequences available in the GenBank database.

A stool sample of a healthy child in Yunnan, China, was inoculated into human embryonic lung diploid fibroblasts (KMB17) and propagated in up to three passages. The sample induced a cytopathic effect (CPE), was stored at −80°C, and was named strain K292/YN/CHN/2013. The viral RNA was extracted from cell culture supernatants using the AxyPrep body fluid viral DNA/RNA miniprep kit (Axygen, Union City, CA). Then, the partial VP1 gene was amplified using the primer pairs 222 and 224 (5) with a PrimeScript one-step reverse transcription-PCR (RT-PCR) kit v.2 (TaKaRa, Dalian, China). The primers used in this study were designed using the primer-walking strategy (6, 7), shown in Table 1. The Tsingke Sequencing Company (Kunming, China) sequenced the positive PCR products using an ABI 3730XL automatic sequencer (Applied Biosystems, Foster City, CA). In order to resolve the alignments into a consensus, DNAStar 6.0 (DNAStar, Inc., Madison, WI) was used to assemble the raw sequences using default parameters. Then, the conflicting positions were inspected manually and amended where manual inspection of the corresponding positions in the chromatograms showed a single peak. The average depth was 3×, based on the read number and length.

**TABLE 1:** Primers used for RT-PCR and sequencing of the human enterovirus C99 strain K292/YN/CHN/2013 genome

Primer name^*a*^	Sequence (5′→3′)	Nucleotide position
222	CICCIGGIGGIAYRWACAT	2969–2951
224	GCIATGYTIGGIACICAYRT	1977–1996
EV991F	TTAAAACAGCTCTGGGGTTG	1–20
EV992R	CTGAAGTCTGGGCACGCGCT	2433–2414
EV992F	GCATGCGTCACTATATTGGA	2741–2760
EV998R	CTCCGAATTAAAGAAAAATT	7428–7409
EV991r	CCCAGGTAGTGATAGAAC	1267–1249
EV992r	GTAATAGGGATTTGTGTA	1707–1696
EV993r	CCATGGTGCAGCTAGACTGT	2270–2251
EV995f	TTGATCAAATTGCCAGAT	3549–3566
EV996f	AGGCCATACAACTGATGGA	5001–5019
EV996r	TTTAAGAGCTTCAAACCA	6751–6734
EV998f	AACCCAGGTGTTGTTACC	6602–6619
EV995r	CAGTAAATCATTGATGCAC	5200–5182

a F (forward) and R (reverse) indicate the orientation of each primer. Primers named with capital (F, R) and lowercase (f, r) letters were used to perform RT-PCR and sequence the amplicons, respectively.

**FIG 1:**
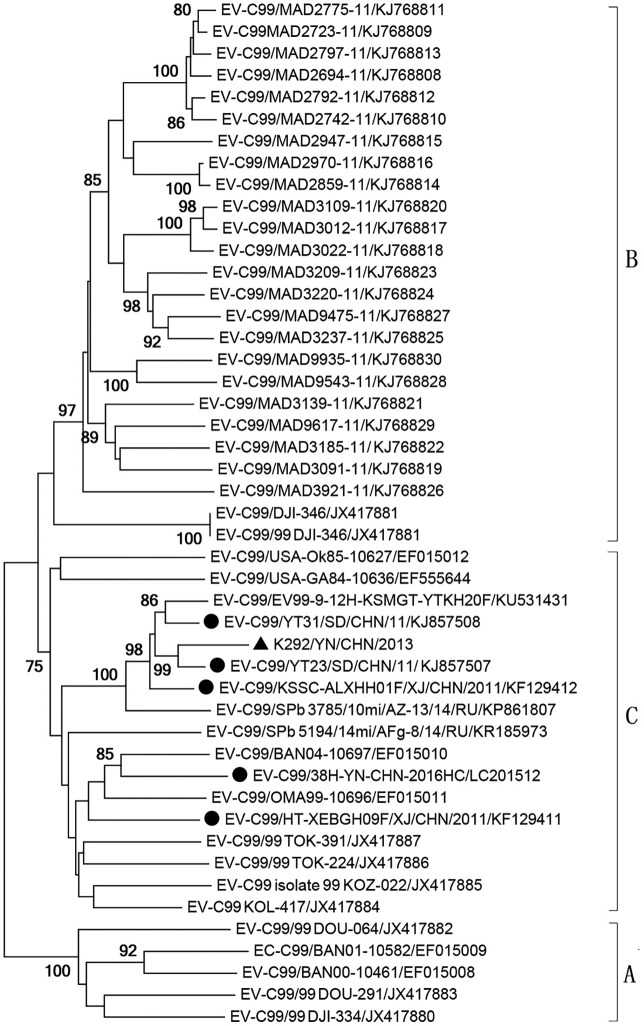
Phylogenetic analysis of EV-C99 based on complete VP1 sequences. The tree was created using the neighbor-joining method with the Kimura two-parameter model of nucleotide substitution in MEGA v.6.06, with a bootstrap value of 1,000. The tip/taxon labels include the strain names and GenBank accession numbers. The triangle indicates the strain isolated in this study, and circles indicate other Chinese isolates.

Geneious Basic 5.6.5 software was used to analyze nucleic acid and protein sequence alignments. The viral genome sequence of the strain K292/YN/CHN/2013 was 7,453 nucleotides (nt) in length, excluding the poly(A) tail. The open reading frame (ORF) of the strain encodes the structural protein region P1 (2,649 nt) and the functional protein regions P2 (1,722 nt) and P3 (2,259 nt). The sequence was flanked by a 3ʹ UTR (71 nt) and a 5ʹ UTR (752 nt). The contents of A, U, G, and C were 30.2%, 24.9%, 22.3%, and 22.7%, respectively, with a GC content of 44.9%. All complete genome sequences of EV-C99 available in GenBank were identified using BLAST. The sequences were aligned using the ClustalW multiple alignment method. The strain shared 81.2 to 92.2% and 87.3 to 93.1% nucleotide similarity with other Chinese strains in the complete VP1 gene and the complete genome, respectively. The isolate was defined as an EV-C99 strain according to the enterovirus type demarcation criterion (strains with >75% nucleotide or >88% amino acid homology with VP1 sequences belong to the same serotype) (7). The strain K292/YN/CHN/2013 is assigned to genotype C, according to the results of the phylogenetic analyses (Fig. 1). In addition, similarity plot and bootscanning analysis was performed using Simplot v.3.5.1; the K292/YN/CHN/2013 strain revealed intraserotypic genetic recombination events.

### Data availability.

The complete genome sequence of K292/YN/CHN/2013 has been deposited in GenBank under the accession no. KT946713.
